# Characterizing cassava farmer typologies and their seed sourcing practices to explore opportunities for economically sustainable seed business models in Rwanda

**DOI:** 10.1177/00307270211045408

**Published:** 2021-11-30

**Authors:** Fleur Kilwinger, Samuel Mugambi, Rhys Manners, Marc Schut, Silver Tumwegamire, Athanase Nduwumuremyi, Sylvie Bambara, Marthe Paauwe, Conny Almekinders

**Affiliations:** 1 Knowledge, Technology and Innovation (KTI), 4508 Wageningen University, Wageningen, The Netherlands; 2 105528 International Institute of Tropical Agriculture (IITA), Rwanda; 3 Rwanda Agriculture and Animal Resources Development Board (RAB), Rwanda; 4 SPARK, Rwanda

**Keywords:** Seed-sourcing strategies, adoption, *manihot esculenta*, seed tracing, tailored approaches

## Abstract

The overdependency on local cassava varieties and informal seed sources by farmers in Rwanda has contributed to the spread of cassava viral diseases. The use of improved planting materials made available through formal seed sources, that assure seed quality, is one way to prevent future disease outbreaks. In order to increase the availability of, and farmers access to, such materials there is increasing interest to develop seed business models. This study aims to understand seed sourcing practices of different farm typologies to inform the development of tailored seed business models. A total of 390 farmers were interviewed and the collected data was analyzed into clusters, resulting in seven farm typologies. Seed sourcing strategies, seed replacement dynamics and purchasing behavior of these typologies were explored via a seed tracing study. We find that more commercial oriented farmers have better access to formal seed sources. Nevertheless, the majority of farmers in all typologies accessed new varieties and quality cassava seed via informal channels. At both formal and informal sources, cash investments in seed were mainly made by the categories of better-off farmers, and were one-time investments to acquire a new variety. Based on farmers current seed sourcing practices, clarifications on the differences between farmers and their willingness-to-pay, the roles of seed degeneration, cost-benefit analysis, value propositions and profit formulas seem important requirements for the further development of viable cassava seed business models. We conclude that tailoring seed business models can have a high potential as it acknowledges differences among farmers, but that careful coordination is needed to ensure that one approach or intervention does not contrast with and/or undermine the others.

## Introduction

### Cassava production and its challenges in Rwanda

Cassava (*Manihot esculenta*) is a major staple crop in sub-Saharan Africa with over 200 million people depending on it for a large part of their calory intake (Manyong et al., 2000). The crop is gaining further economic importance as a raw material for the industrial processing of foods, ethanol, and starch. Cassava Mosaic Disease (CMD) and Cassava Brown Streak Disease (CBSD) are currently the most threatening biotic stresses to cassava production in East and Central Africa (Alicai et al., 2007; Legg et al., 2001; Tumwegamire et al., 2018). The two diseases cause devastating effects on root quantity and quality, with field and storage losses ranging from 30% to 100% (Kawuki et al., 2016; Okonya et al., 2019; Patil et al., 2015). Both diseases spread via a whitefly vector (*Bemisia tabaci*) and the use and exchange of infected planting material^1^ (Legg et al., 2011). This means that farmers’ use of local susceptible varieties and recycling of stem cuttings from the previous crop can aggravate the impact of the diseases. The introduction of resistant varieties and availability of clean planting material is therefore of high importance (Night et al., 2011).

CBSD incidence peaked in Rwanda between 2010 and 2015, and severely threatened food security. To cope with the situation, the government of Rwanda imported clean planting materials (stem cuttings) of CBSD resistant varieties from Uganda for distribution to smallholder farmers. Cassava variety development and production of quality clean planting material in Rwanda is, like in many developing countries and for other vegetatively propagated (staple food) crops (VPCs), mainly in the hands the government and development organizations. Also typical for VPCs in developing countries, governments and non-governmental organizations (NGO) often subsidize multiplication and buy the planting material to distribute among farmers (Bentley et al., 2018; Rachkara et al., 2017; Tripp and Rohrbach, 2001).

Studies that critically looked at such funding-driven strategies have observed negative consequences (Sperling et al., 2008; McGuire and Sperling et al., 2008). For example, in the short-term farmers may waste scarce resources like land and labor when provided with maladapted varieties. In the long-term, having continuous access to free or subsidized seed supply may easily create dependency among farmers and disrupt the market for seed, hindering the emergence of viable commercial seed enterprises (Rohrbach et al., 2005; Tripp and Rohrbach, 2001). Consequently, seed system research in Sub-Saharan Africa is increasingly focused on developing economically sustainable seed business models that can carry forward the supply of improved healthy planting materials after such an intervention (Donovan et al., 2021; Rachkara et al., 2017; Sperling et al., 2013).

### Commercial seed business models for vegetatively propagated crops

A business model can be defined as a representation of how an organization views, creates, distributes, and captures value for itself (via a profit formula), and for users (defining the value proposition). This aspect is often neglected in innovation efforts, which instead tend to focus on the goods or services themselves, rather than on management and the creation of value (Campos, 2021). Although business models are often associated with profit making, this is not necessarily true. It can be argued that non-profit organizations, including those focusing on agricultural development, already run a busines as they are under pressure to innovate to meet the continually evolving needs of their beneficiaries (Campos, 2021).

Nevertheless, moving from aid-based systems towards commercialized seed systems is considered by many as a more effective and economically sustainable (CtEH, 2021; Rachkara et al., 2017; Tripp, 2003). ‘Commercialized’ refers to activities, like buying and selling seed/planting material, with the intend of making a profit. Such commercialized seed systems can involve multiple actors like decentralized seed multipliers, seed companies, agro-dealers and traders (Sperling et al., 2013). Proposed benefits of commercialized seed systems are improved access to quality seed and high yielding varieties with high market values, higher varietal turnover, increased productivity and food security, reduction of poverty and food imports, increased returns of investments in crop improvement research, and attraction of private sector investments (Barker et al., 2021; CtEH, 2021; Maredia et al., 2019).

Private sector investments for the improvement of cassava in Rwanda has so far been low. There are many reasons why VPC seed systems are generally less attractive to commercialize by private organizations than ‘true seed’ crops: their genetic complexity often complicates breeding, the propagation material of VPCs is usually bulky, perishable, and easily carries pests and diseases (Bentley et al., 2018; McGuire and Sperling, 2016; Thiele, 1999), and because of the clonal nature, planting material remains ‘true to type’ after multiplication, providing a low incentive for farmers to become frequent buyers (Almekinders et al., 2019).

When developing economically viable seed business models, a solution needs to be found for the challenges and bottlenecks that are related to these characteristics. Because the material remains true to type (lack of a clear value proposition), there is no incentive to invest (distorting the profit formula) in new planting material, unless the materials provided by the seed business offer a clear advantage over farm-saved seed. According to Tripp (2003), this advantage can be in the form of access to new varieties or clean seed and should translate into increased productivity benefits. This also implies that information on demand for different varieties/planting materials is available (Barker et al., 2021). Better insights into demand, farmers’ seed sourcing strategies, replacement dynamics, purchasing behavior, and the underlying motivations and differences among farmers in these issues, are required.

### Understanding and predicting demand

Earlier research has shown that access to, and demand for, planting material varies among farmers, even within informal seed systems (e.g. Coomes et al., 2015; Kilwinger et al., 2020; McGuire, 2008). Furthermore, a fluctuating pattern can be observed with the demand being low after a ‘good’ season and high after a ‘bad’ season that seed businesses would have to deal with (Almekinders et al., 2019). Several case studies have shown that farmers are willing-to-pay for VPC planting material in Sub-Saharan Africa (e.g. Bartle and Maredia, 2019; Boadu et al., 2019; Maggidi, 2019) and that commercial seed sectors are emerging in Sub-Saharan Africa (e.g. Bentley et al., 2020; Gibson et al., 2009; Namanda et al., 2011), though there is little detail of the type of farmer who is prepared to pay and under which conditions (Rachkara et al., 2017).

The heterogeneity among farmers, their farms, and farming practices creates the risk that a “one-size-fits-all” strategy or model would favor and appeal to only a specific group of farmers. One-size-fit-all strategies might be easier to scale, but may be inefficient to achieve adoption at scale compared to more nuanced ‘tailored approaches’. Those are data-driven approaches that incorporate farmers’ diversity in scaling strategies, can result in higher adoption rates by meeting farmers’ diverse needs and capacities, and support greater development impact and (Hammond et al., 2020).

The objective of this study is to develop farmer typologies and get insights in their cassava seed sourcing practices, seed replacement dynamics and purchasing behavior. This was done by linking farmer typologies to a seed tracing study. The outcomes can support the development of tailored seed business models. Furthermore has it been demonstrated it is important to understand social and cultural factors that shape seed exchange as they influence disease spread and populations (Delêtre et al., 2021; Garrett, 2021). Next to a contribution to the development of tailored business models for cassava seed, the study is a methodological contribution by linking farmer typologies (Hammond et al., 2020) with seed tracing. Lessons learned from the Rwanda case study can inform the use of this approach in other countries and for other innovations.

## Methods

### Study design

We used a household survey and a seed tracing study to characterize the cassava seed system in Rwanda. The household survey was conducted to cluster cassava farmers into different typologies. For the household survey we used an adaptation of the Rural Household Multi-Indicator Survey (RHoMIS) tool (Hammond et al., 2017). A sub-sample of farmers from the household survey was selected for a seed tracing study. Data from the seed tracing was then linked to the household survey data to identify patterns of seed movement among different stakeholders and farmer types.

### Site selection and sampling

For the household survey we used an adaptation of the Rural Household Multi-Indicator Survey (RHoMIS) tool (Hammond et al., 2017). The RHoMIS tool is designed to rapidly characterize a series of standardized indicators that cover the spectrum of agricultural production and market integration, nutritional status and food security, gender equity and poverty. It also enables rapid characterizations of both farm practices and farm performance, thus allowing one to group and assess farming households around a variable of interests; in our case access to quality cassava planting material. Because of its standardized indicators, RHoMIS also allows comparison across geographies and monitoring across time, which is useful for multi-country or region and time-bound interventions.

**Figure 1.**
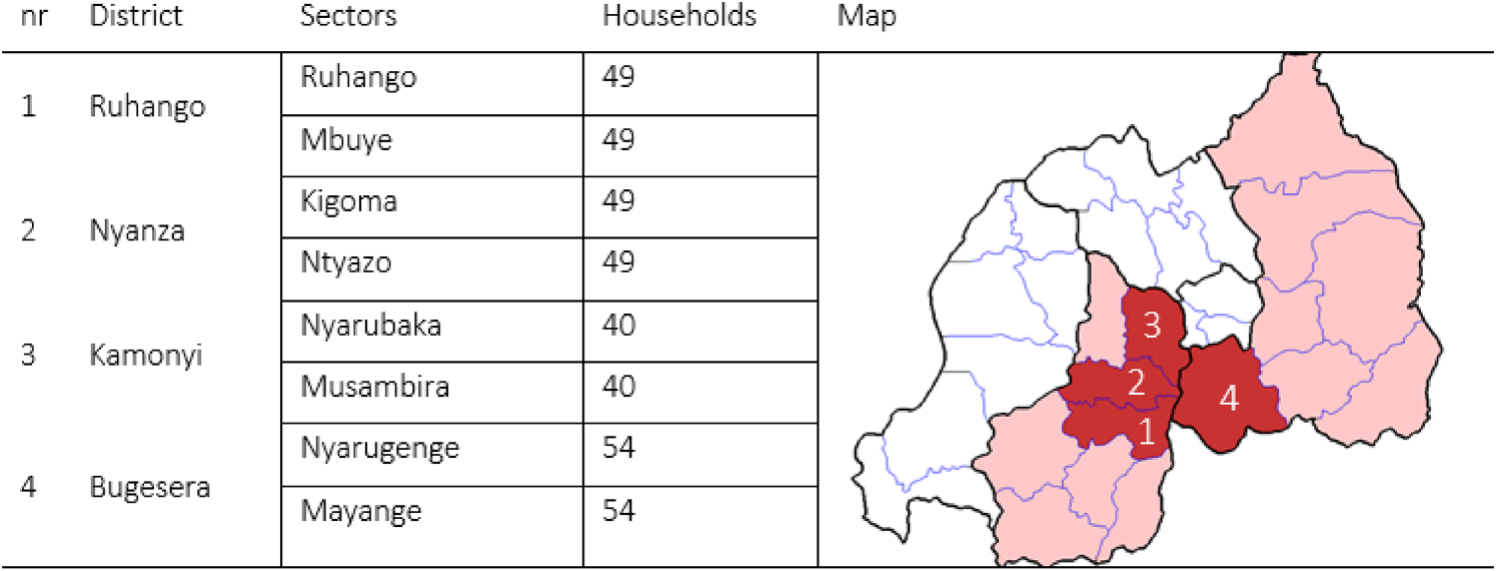
Number of households interviewed per district and sector. Districts are shown on the map of Rwanda. (image modified from source: Https://nl.wikipedia.org/wiki/Bugesera).

In total, 390 farming households were surveyed in four districts of Rwanda, where cassava production is most prevalent: Ruhango, Nyanza, Kamonyi, and Bugesera. Data was collected in November and December of 2019. Using the number of farming households in each district as a baseline, a sample size sufficiently large to detect the diversity of the farming households, was calculated with a 95% confidence interval and 5% margin of error. The resultant minimum sample for each district was divided proportionally across two randomly selected sectors of the same district (Figure 1). The determined sector sample sizes were then evenly distributed across four random villages with the district.

Following Hammond et al. (2020), enumerators were instructed to start at the center of the selected villages. Then, a random direction and number (*n*) were generated. The enumerator had to select the *n*th house based on the generated number, in the generated direction. Following completion of the interview, enumerators returned to the center of the village and this process was repeated until the predetermined number of households to be surveyed in the village was reached. Open Data Kit (ODK) forms were pre-installed on Android tablets to collect the data.

### Developing farmer typologies

For the survey, more than 60 variables were selected as potential explanatory variables for farming household diversity, in particular in relation to the households’ access to quality cassava seed. These variables included demographic, agronomic, economic and cassava-specific variables. This list was reduced through knowledge of the sites, understanding of seed systems, and logical analysis, to 17 variables (Table 1). Data for these variables were examined to identify missing data and outliers. Two multivariate statistical steps were applied to develop our farmer typologies: i) reducing the dimensionality of the data through the application of principal component analysis (PCA); and ii) cluster analysis for partitioning into clusters.

**Table 1.** Variables used for farm typology development.

Variable	Unit
*Household*	
Household Head Age	Age
Household Head Sex	Male/Female
Household Head Marital Status	Categorical
Years on Farm	Years
*Assets*	
Radio Ownership	Yes/No
Asset Count	Number of assets
*Farm Structure*	
Inorganic Inputs Count	Number of inputs
Total Livestock Units	Number of TLUs
Cassava Production Sold	Yes/ No
Positive Farm Changes	Custom Score
*Economic*	
Total Crop Income	USD
Access to Finance	Yes/No
Credit Received	Yes/No
*Seed Systems*	
Cassava Seeds Sold	Yes/No
Seed Multiplication as a Business	Yes/No
Improved Varieties Used	Yes/No
Improved Varieties Count	Number of varieties

#### Principal component analysis

To reduce the dimensionality of the dataset, we employed principal component analysis (PCA; Jolliffe, 2002). PCA has been widely employed in typology generation in farming systems (e.g. Alvarez et al., 2018; Kuivanen et al., 2016; Lopez-Ridaura et al., 2018). The analysis was performed using the *ade4* package of R (Dray and Dufour, 2007; R Core Team, 2019). We retained relevant principal components through application of two criteria: i) Scree test; and ii) that the PCs had an eigenvalue greater than 1 (Kuivanen et al., 2016). Application of these criteria resulted in the retention of 5 principal components (Table 2).

**Table 2.** Eigenvalues and variance (individual and cumulative) of the five principal components from PCA.

Principal Component	Eigenvalue	Variance (%)	Cumulative Variance (%)
1	3.8	19	19
2	2.4	12	31
3	2.2	11	42
4	1.9	9	51
5	1.6	8	59

#### Cluster analysis

We used the partitioning around the medoids method for clustering (PAM; Reynolds et al., 1992). PAM identifies a medoid observation (in this case farm) that is most representative of a cluster and seeks to reduce the dissimilarity of other observations to that medoid (Reynolds et al., 1992). To develop the dissimilarity matrix for PAM, we followed Hammond et al. (2020) and applied the Gower method (Gower, 1971), which allows for inclusion numerical, ordinal, and categorical data types. To determine the optimal number of clusters to be retained, we reviewed the silhouette width metric, with the silhouette width being highest for 7 clusters. The inherent structure of the clusters was then evaluated (e.g. mean and modal values for each variable and cluster).

Testing for the significance (α 0.05) of variables between clusters was done using the chi-squared test for binary variables, one-way ANOVA for parametric continuous variables, and Kruskal Wallis for non-parametric continuous variables. When a significant difference was identified via Kruskal Wallis, further comparison between groups was done via Mann-Whitney U tests with an adjusted alpha of 0.002. To further characterize the clusters they were assigned a descriptive name based upon on the variable(s) that most distinguished them from other clusters.

### Cassava seed tracing

An improved variety, NASE14, introduced in response to CBSD outbreak, was traced via snowball sampling (Goodman, 1961). Data was collected in January and February 2020. The seed tracing study started with a sub-sample of 61 farmers selected randomly from the household survey respondents with a random number generator in Excel. Farmers were selected form the same districts, Ruhango, Nyanza, Kamonyi, and Bugesera, as the respondents of the typology survey. All the participants in this sub-sample were interviewed about their cassava seed sourcing practices. If the farmer grew NASE14, we traced from where the farmer obtained this variety until a formal actor such as a seed multiplier, NGO or RAB was reached. This resulted into an additional 11 interviews with farmers that exchanged NASE14 seed and who provided links between the surveyed farmers and formal actors. The data from the typology analysis were linked to the tracing data.

To visualize transactions, the data from the seed tracing study was further analyzed following the protocol for ‘seed tracing’ (Kilwinger and Buddenhagen, 2021). This resulted in a ‘node-list’ and an ‘edge list’. The nodes represented the interviewed actors. Additional information, such as the type of actor and demographic information were included in the node list. The edge list was based on transactions of NASE14 planting material between the actors (nodes). Similarly, additional information on the transactions such as quantity, frequency, and transaction type was included in the edge list. Using this node and edge list, a network graph was created in the Excel add-in ‘NodeXL’.

In addition, a list of seed multipliers provided with NASE14 seed was obtained from Rwanda Agricultural and Animal Resources Development Board (RAB) (the national institution mandated to release varieties and provide the first clean seed). This resulted in the identification of 16 seed multipliers who were then asked about their selling of cassava stems.

## Results

### Cassava farmer typologies

The 390 farmers interviewed during the RHoMIS survey were grouped into 7 clusters using principal component analysis (Table 3). There seemed no geographic aspect to this response as farmers in all clusters were scattered over the sample areas.

**Table 3.** Demographic and cassava farming information of the 7 generated farmer typologies via household survey data (n = 390). All presented values are means or proportions. Letters indicate statistical differences (α 0.05) tested with the chi-squared test for binary variables, one-way ANOVA for parametric continuous variables, and Kruskal Wallis and Mann-Winey U for non-parametric continuous variables. Variables that were not significant or violated assumptions for statistical testing are indicated as ‘ns’.

Cluster (n)	1 (39)	2 (138)	3 (51)	4 (76)	5 (34)	6 (19)	7 (33)	Total
HH head sex is male (%)	100^a^	100^a^	90^a^	95^a^	0^b^	0^b^	15^b^	60
HH head age	50^b^	45^a^	41^a^	43^a^	51^b^	54^b^	59^c^	47
HH head marital status is married (%)^ns^	97	93	90	91	12	16	18	61
HH head has no formal education (%) ^ns^	46	54	67	45	65	84	85	58
HH members	5.43^ab^	5.44^a^	5.03^ab^	5.47^a^	4.52^ab^	4.10^ab^	4.12^b^	5.13
Females in HH are involved indecision making (%)	52^a^	53^a^	62^b^	53^a^	89^c^	9°^c^	89^c^	70
Farm size (ha)	0.69^ad^	1.02^a^	0.33^bc^	1.22^a^	1.17^a^	0.28^bcd^	0.64^ac^	0.76
Years on farm	28^bc^	22^c^	21^c^	22^c^	30^b^	33^ab^	40^a^	28
Market travel time (minutes)	41^a^	62^ab^	74^b^	64^ab^	52^ab^	56^ab^	65^ab^	59
Number of assets household owns/has access to	2.2^ab^	2.4^a^	0.7^c^	2.5^a^	2.0^ab^	1.7^b^	0.3^c^	1.7
Number of inorganic inputs used by household last year	1.5^a^	1.4^a^	0.4^b^	1.5^a^	1.0^ab^	0.8^ab^	0.4^b^	1.0
Total livestock unit (TLU)	0.94^ab^	0.84^a^	0.76^ab^	1.09^a^	0.79^ab^	0.46^ab^	0.44^b^	0.76
Livestock diversity (types of livestock owned by household) ^ns^	1.8	1.6	1.5	2.0	1.3	1.0	1.4	1.5
Crop diversity (number of crops grown by household)^ns^	3.7	3.5	3.4	3.9	3.7	3.3	3.6	3.6
Total crop income (RWF^2^)	110^bc^	231^cd^	65^ab^	298^d^	183^cd^	104^bcd^	27^a^	107
Number of positive farm changes last 4 years	2.1^ab^	2.4^a^	1.7^ab^	2.8^a^	2.1^ab^	1.5^ab^	1.2^a^	2.0
Most important crop is cassava (%)^ns^	71.8	71.7	66.7	72.4	73.5	57.9	51.5	69.0
Farm area dedicated to cassava (%)	41^ab^	53^a^	41^b^	50^ab^	54^ab^	51^ab^	39^b^	47
Total area cassava (ha)	0.31	0.59	0.14	0.69	0.65	0.15	0.26	0.48
Cassava yields (kg/ha) ^ns^	4016	3138	4471	3925	3271	3698	3973	3785
Share of cassava production sold (%)	29^abc^	34^ab^	25^c^	45^a^	34^abc^	16^bc^	13^c^	28
Has enough quality cassava seed (%)	31^abcd^	46^d^	28^c^	37^abcd^	50^bd^	47^abcd^	24^ac^	39
Aware of benefits certified seed (%)	92^ab^	80^bc^	77^cd^	92^a^	82^abcd^	80^abcd^	64^d^	82
Uses improved seed (%)	38^a^	36^a^	10^b^	40^a^	27^ab^	15^ab^	15^b^	31
Pays for quality cassava seed (%)	26^a^	30^a^	31^a^	53^b^	27^a^	16^a^	18^a^	32
Distributes quality cassava seed (%)^ns^	33	49	37	55	41	36	33	44
Sells cassava seed (%)^ns^	80	97	96	98	97	89	87	94
Wants to be a seed multiplier (%)	0^a^	100^b^	86^c^	97^b^	100^b^	0^a^	21^d^	76
Has access to finance for cassava farming (%)	36^ab^	45^b^	35^ab^	68^c^	32^ab^	37^ab^	24^a^	44
Has attempted to get credit for cassava farming (%)	5^ab^	0^c^	14^ab^	100^d^	12^ab^	21^b^	3^a^	25

The largest category of farmers were clustered as type 2 (35%) (Table 4). These farmers were interpreted to be ‘average cassava farmers’. They are generally male-headed households with relatively high levels of assets and relatively large farm sizes, but relatively poor cassava productivity (Table 3). The second largest cluster was type 4. We named this group ‘professional farmers’. They are generally male-headed households, and have the most assets and livestock and the largest farm sizes. The household heads were relatively highly educated and attained the highest revenue from their crops out of all the typologies.

**Table 4.** Descriptions of the farmer typologies and their representativeness (%) in the whole sample (n = 390).

Type	Description of farmer typology	%
1	**Older farmers** Male-headed households, married, primary education, older household head, close to market, high asset count, high use of inputs, no interest in being a seed multiplier.	10%
2	**Average cassava farmers** Male-headed households, married, no education, larger farm size, relatively low yield, high asset count, interested in being a seed multiplier.	35%
3	**Small-scale farmers** Male-headed households, married, no education, small farms but with high yields, younger household heads, female partners more often involved in decision making, low asset count, low use of inputs, little use of improved cassava seed, low income from crops, sells relatively little cassava.	13%
4	**Professional cassava farmers** Male-headed households, married, primary education, largest farms, highest asset count, high use of inputs, high use of improved cassava seed, pays for quality seed, access to finance for cassava farming, highest income from crops.	19%
5	**Better-off female-headed households** Female-headed households, widowed, no education, large farm, high asset count, medium use of inputs, relatively low yields, higher access to quality seed, interested in being a seed multiplier, high income from crops.	9%
6	**Small-scale female-headed households** Female-headed households, widowed, no education, smallest farms, medium assets, have access to quality seed, sells relatively little cassava, no interest in being a seed multiplier.	5%
7	**Elderly poor female- headed households** Female-headed households, widowed, no education, older household head, more years on farm, medium farm size, fewest assets, least use of inputs, sell smallest part of their cassava, have the lowest crop income.	8%

Farmers clustered in type 1 and 3 are both generally male-headed households who mainly differ based on age of the household head and farm size. With a mean of 50 years, the household heads of typology 1 were significantly older than those of other male-headed typologies. With a mean of 41 years, the household heads of type 3 were the youngest of all developed typologies. Type 1, named ‘older farmers’, had higher education, more assets, and used more farm inputs. Type 3, named ‘small-scale farmers’ had the smallest farms, fewest assets and used the least inputs of the male-headed typologies. Despite having less resources available, the productivity of these households is high, which is not uncommon for small farms of young households.

The last three types 5, 6 and 7 are all female-headed households (mostly widowed, and some divorced or single). Female-headed households together made up for 23% of the households surveyed. Type 5, ‘better-off female-headed households’ had larger farm sizes, perceived themselves to have better access to quality cassava seed, and had a relatively high asset count as well as crop income. Type 6 ‘small-scale female-headed households’ are characterized by very small land sizes. Type 7 ‘elderly, poor, female-headed households’ seemed to be in the most vulnerable position: the household heads are older, they have the least assets, use the least farm inputs, reported the lowest crop income, and grow the largest share of cassava for home consumption. The average reported crop income of type 7 (RwF^2^ 27,000) is about a quarter of the reported crop income of type 6 (RwF 104,000), even though they have on average twice as much land available (Table 3).

### Seed sourcing practices

Farmers in the household survey (n  =  360) grew between 1 and 5 cassava varieties with a mean of 1.84 (SD  =  0.74). In total 46 varieties were grown by farmers. The majority (28) were local varieties, also known as landraces, of which many (20) were grown by 1% or less of the farmers (Annex 1). Of the remaining 18 varieties, 16 were introduced in Rwanda since 1975, and of 2 the introduction date is unknown. Via a list provided by RAB, 10 of those varieties were identified as improved and officially released since 2005. NASE14 was the variety grown by most farmers (51%). Besides its dual resistance for CMD and CBSD, farmers are fond of the variety because it has multiple purposes. The sweet taste makes it usable in fresh form (boiled roots are a common local dish), and because it is heavy, it is suitable for flour processing as well. These traits make the variety fetch a high market price, providing the household with both food and income.

NASE14 was officially released in 2018 (after being tested at trials), but had been with farmers since introduction from Uganda in 2015 (IITA, 2015). When preferred by farmers, varieties tend to spread from demonstration plots before being officially released. For example, Macadamia, the most grown improved variety after NASE14 (grown by 44%), was tested on research stations around 2009. It never got formally released until 2021 because researchers found its dry matter content too low. Farmers nevertheless kept growing the variety because of its early bulking and multiple end uses.

The majority of the farmers (85%) grew one or more varieties introduced after 2005 (Table 5). Of the farmers classified as older farmers (type 1) the fewest grew improved varieties (77%) and of the professional farmers (type 4) the most (96%). Slightly less than half of all the farmers (48%) grew only improved varieties. Of the professional farmers most farmers grew only improved varieties (57%) and of elderly poor FHH (type 7) the least (33%). In general, 26% of the farmers grew both improved and local varieties. A few farmers grew only local varieties (6%). Of the better-off FHH (type 5) most farmers grew only local varieties (12%) whereas none of the professional farmers grew only local varieties. The varieties of which the original source was traced were mainly informal, 67% came from a fellow farmer. The majority of farmers started growing improved varieties between 2014 and 2019 with the highest number of farmers reporting 2017 as the year they originally sourced an improved variety.

**Table 5.** The percentage of farmers per typology growing improved cassava varieties, perceived themselves as having access to quality seed, and the sources they use to access new varieties and quality seed (n = 390).

	Typology ‡							
Variable	1	2	3	4	5	6	7	Total
Number of cassava varieties grown	1.85	1.78	1.86	1.99	1.62	1.79	1.94	**1.84**
Grows one or more improved varieties	77%	80%	84%	96%	85%	89%	85%	**85%**
Grows NASE14	49%	44%	49%	62%	59%	53%	48%	**51%**
Grows only improved varieties	36%	51%	51%	57%	53%	37%	33%	**48%**
Grows one or more local varieties	36%	28%	31%	29%	32%	32%	36%	**31%**
Grows improved and local varieties	28%	21%	29%	29%	26%	26%	33%	**26%**
Grows only local varieties	10%	8%	4%	0%	12%	5%	6%	**6%**
Sources new varieties from fellow farmers §	90%	69%	100%	59%	67%	100%	50%	**67%**
Sources new varieties from seed multipliers §	0%	22%	0%	20%	20%	0%	50%	**19%**
Sources new varieties from RAB §	10%	7%	0%	16%	7%	0%	0%	**10%**
Sources new varieties from NGO projects §	0%	2%	0%	5%	7%	0%	0%	**3%**
Has access to quality cassava seed	31%	46%	27%	37%	50%	47%	24%	**38%**
Knows where to access quality cassava seed	28%	13%	25%	30%	21%	11%	9%	**20%**
No access nor knows where to access quality cassava seed	41%	41%	47%	33%	29%	42%	67%	**43%**
Sources quality cassava seed from own farm	28%	32%	24%	18%	32%	26%	9%	**24%**
Sources quality cassava seed from fellow farmers	8%	25%	16%	22%	26%	26%	18%	**20%**
Sources quality cassava seed from seed multipliers	0%	2%	0%	4%	0%	0%	0%	**1%**
Sources quality cassava seed from RAB	3%	1%	0%	3%	0%	0%	0%	**1%**
Sources quality cassava seed from cooperative	0%	0%	0%	1%	0%	0%	0%	**0%**
Would source quality cassava seed from fellow farmers	18%	11%	16%	24%	18%	11%	9%	**15%**
Would source quality cassava seed from Seed multipliers	8%	4%	6%	9%	3%	5%	0%	**5%**
Would source quality cassava seed from RAB	5%	1%	2%	8%	3%	0%	0%	**3%**
Would source quality cassava seed from cooperative	5%	1%	0%	4%	0%	0%	3%	**2%**

‡ Farmer typologies: 1  =  Older farmers; 2  =  Average farmers; 3  =  Small-scale farmers; 4  =  Professional farmers; 5  =  Better-off FHH; 6  =  Small-scale FHH; 7  =  Elderly, poor FHH.

§ Data used from seed tracing dataset with different sample sizes, farmers could mention the original source for more than 1 variety resulting in 159 observations : 1) n = 10; 2) n = 54; 3) n = 5; 4) n = 64; 5) n = 15; 6) n = 5; 7) n = 6.

On average, 38% said that they have access to high quality cassava seed. ‘Quality’ was not defined during the interviews. Thus, if farmers said that they have access to quality cassava seed, this was according to their own perceptions^3^. Among all types, most farmers who claimed that they did not have access to quality seed, also did not know where they could access high quality material. Only 20% said they knew sources with quality material available. Thus, in general 43% of the farmers did not have access to quality material nor knew where they could access it. The perception of having access to quality cassava seed seemed more variable among farmer typologies compared to access to improved varieties. In particular many elderly, poor female-headed households (type 7) perceived a lack of access (67%), especially compared to better-off female-headed households (29%).

Most farmers who said they have access to quality seed perceived informal sources (their own farm and fellow famers) as the main source. Professional cassava farmers were the most likely to access their seed from formal sources. A small percentage of professional cassava farmers accessed quality seed from seed multipliers (4%), cooperatives (1%) and RAB (3%). An even smaller percentage of older and average cassava farmers (types 1 and 2) accessed quality seed from formal sources. Other typologies, small-scale farmers and all female headed households (type 3, 5, 6 and 7), said they did not use formal sources to access quality seed, and only a few ( ≤ 6%) perceived them as potential sources. They perceived their own farm and fellow farmers as sources for quality seed

### Seed replacement dynamics and purchasing behavior

In total, 52 farmers were interviewed that grow NASE14 to identify the original source of the variety. Of these farmers 41 participated in the household survey and were assigned to a typology. Through snowball sampling 19 additional farmers, that provided surveyed farmers with NASE14 seed, were identified. Of these farmers 11 could be reached for a follow-up interview to further trace where they sourced NASE14. The majority of the farmers identified in the seed tracing study were male-headed households (types 1 to 4). These were also more abundant in the typology survey (77%) than female-headed households (Table 3).

**Figure 2.**
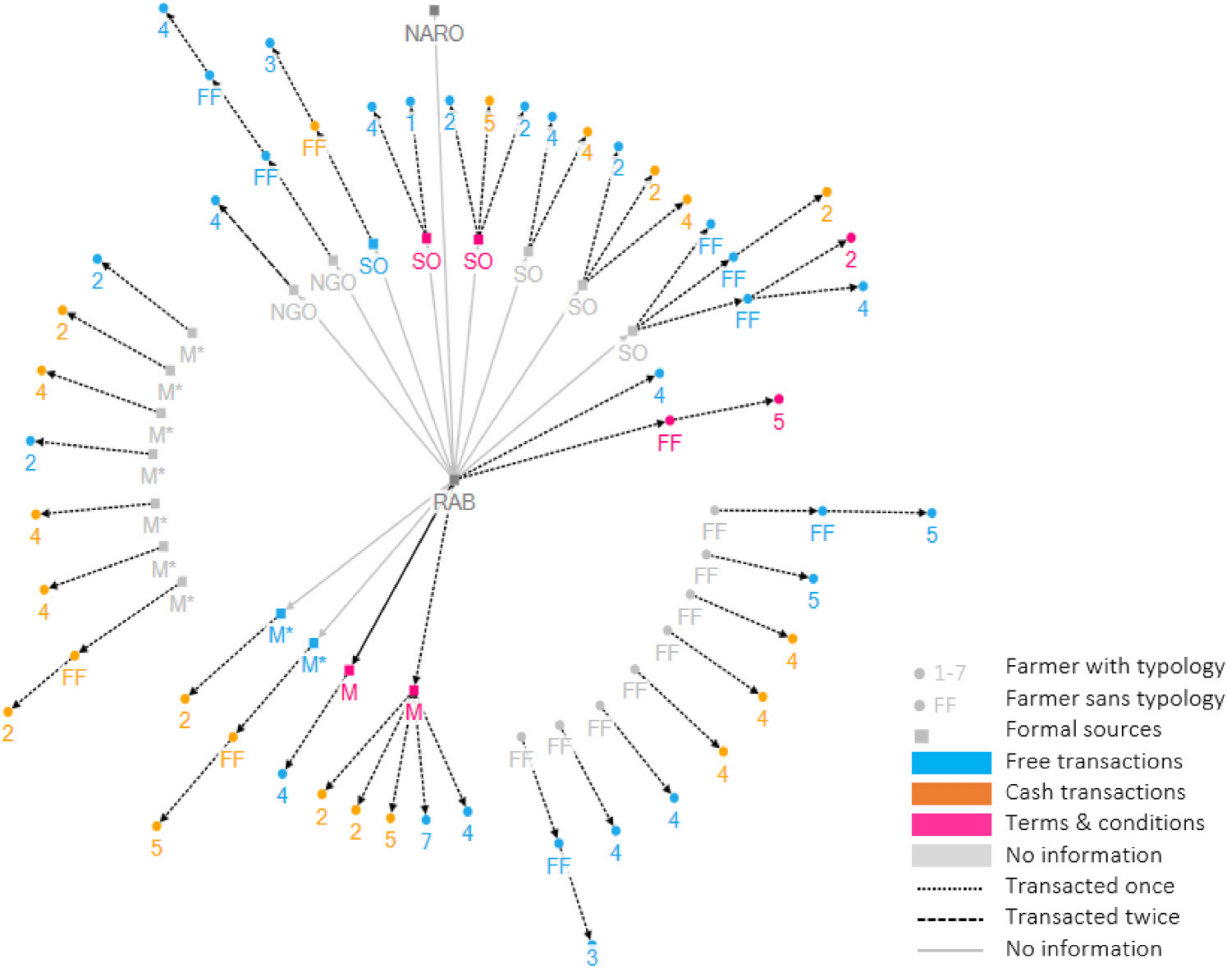
Visualization of tracing NASE14. The arrows indicate exchanges of seed of cassava variety NASE14. The types of actors are indicated using shapes and numbers. Circles represent a farmer assigned to a typology (1–7) or a farmers without assigned typology (FF). Squares represent the formal sources: Sector offices (SO), non-governmental organizations (NGO), NARO, RAB, and official (M) and unofficial seed multipliers (M*). The color of the shapes represents the transaction type: Blue  =  free; orange  =  cash; pink  =  terms and conditions. The line style represents the frequency of transactions: Dotted lines once, dashed lines twice and solid lines more than twice. Grey shapes and lines indicate no information on transaction type and frequency was available.

Farmers obtained NASE14 for the first time between 2014 and 2019 and accessed it from several sources: sector offices (SO), non-governmental organizations (NGO), seed multipliers (M) and fellow farmers (FF) (Figure 2). Several farmers mentioned they sourced seed from a seed multiplier, but these were not officially recognized as such by RAB (M*). SOs, Ms and NGOs got NASE14 planting material from RAB. RAB in turn obtained NASE14 germplasm from the National Agricultural Research Organization (NARO) in Uganda in 2014. Only 2 of the M*s could be identified for a follow-up interview and got NASE14 from RAB. Where the other M*s originally sourced NASE14 could not be traced.

None of the seed multipliers had to pay with cash for NASE14 planting material. Of the 16 seed multipliers officially recognized by RAB, 10 said they obtained the seed from RAB free of charge. They obtained the material between 2014 and 2018. The remaining six multipliers said they had received the seed with certain terms and conditions. This mainly entailed them sharing the same amount of seed as they received from RAB with fellow farmers, as part of RAB's strategy to disseminate new improved varieties. Seed multipliers each obtained between 8000 and 200,000 NASE14 cassava cuttings from RAB. The number of farmers each multiplier provided with seed varied between 20 and 400. Some seed multipliers provided farmers with NASE14 stakes free of charge and others for cash. The number of cash transactions varied between 10% and 100% among multipliers, with a mean of 59%.

Several farmers obtained NASE14 seed from sector offices or NGOs. Three eployers at sector offices could be contacted and they indicated they either got seed from RAB free of charge or under terms and conditions. They reported to have obtained between 1,000,000 and 2,000,000 stems. Sector offices did not further multiply the material, they only distributed. This could be the reason why the volumes of seed provided by RAB were higher compared to the volumes supplied to multipliers. Not from all formal actors information on distributed volumes of seed could be identified, meaning their relative importance could not be further visualized.

In general, 39% of the farmers made a cash investment to acquire NASE14 (Table 6). Only farmers classified as type 2, 4 and 5 made cash investments while acquiring NASE 14 seed, whereas types 1, 3 and 7 obtained it for free regardless of where it was sourced. Compared to the original sources of all varieties, NASE14 was most often sourced from formal sources. Interestingly, Macadamia was originally sourced from fellow farmers by 74% of the farmers, but was more often paid for with cash than NASE14. Similarly, it were type 2,4 and 5 farmers who made those cash investments (data not shown). None of the farmers accessed Macadamia via a government program.

**Table 6.** Varieties original source and transaction type. 188 transactions were recorded from the subsample of 72 farmers.

	All varieties	NASE14	Macadamia
Original source			
Fellow farmer	67%	40%	74%
Seed multiplier	19%	28%	23%
NGO project	3%	4%	3%
GO project	10%	28%	0%
Transaction type			
Free	63%	54%	46%
Cash	34%	39%	54%
In kind/under conditions	3%	7%	0%

With one exception, none of the farmers sourced NASE14 seed off-farm more than once (Figure 2). On the other hand, all the farmers multiplied the NASE14 seed themselves after the first acquisition, and 80% of the farmers went on to share this multiplied seed with fellow farmers. Most farmers (78%) perceived that the quality of the material remained unchanged between them first acquiring it and after they multiplied it, 10% perceived that the quality increased after self-multiplication, and 12% perceived that it decreased.

Nearly all farmers (82%) reported having experienced viral diseases in their cassava fields in the past The severity of the disease infestation on the farm at the worst moment, expressed as % of infected plants, ranged between 10 and 100 with a mean of 52%. The severity of the disease at the time of the interviews ranged between 0 and 40 with a mean of 2%. The practices farmers used to control the disease were: sourcing new seeds (59%), rogueing (41%), and planting improved varieties (39%). The majority of the farmers (90%) said that they had never received training about maintaining the sanitary quality of seed.

## Discussion

### Farmers access to, and investments in, new varieties

More than 85% of the surveyed farmers were growing improved cassava varieties that were formally registered after 2005. A study conducted in 2007 found that 83% of the surveyed farmers in Rwanda grew only local varieties (Night et al., 2011), suggesting a rather effective diffusion and high adoption of improved cassava varieties since then^4^. The majority of farmers started growing improved varieties between 2014 and 2019, making it plausible that adoption was in response to high disease pressure. While farm typologies did not show major differences in the use of improved varieties, the sources through which farmers had accessed them varied. The majority of farmers, mostly, if not entirely, used informal seed sources to acquire new varieties. Farmers labelled as ‘professional farmers’ (type 4) most often used formal sources to acquire new varieties, although the majority of them still reported to use informal sources. Patterns of farmer-to-farmer seed diffusion involving social barriers are common (Almekinders et al., 2020; Coomes et al., 2015; Tadesse et al., 2017), and could have important implications for introduction points of new varieties and other activities. Despite influencing the timing and other acquisition conditions, these social barriers seemed to have little effect on who the variety eventually spread to.

The efforts of RAB and partners to introduce NASE14 seemed successful as half of the farmers grew the variety. Macadamia was also adopted by many farmers and cash transactions to obtain the variety were even more reported than cash transactions for NASE14. This suggests that markets for new cassava varieties emerge naturally when they have desired traits. Nevertheless, none of the formal sources multiplying and/or distributing NASE14 (and likely Macadamia) had to pay for the material they received from RAB. To move away from aid-based seed systems, it seems important to determine at which stage of the seed value chain commercialization should start. Many studies that report the emergence of commercial seed enterprises for vegetative propagated staple crops in Sub-Saharan Africa do not report where and how sellers obtain their material in the first place (see for example Bentley et al.,2020; Rachkara et al., 2017). When prices are based on the actual costs of breeding and early generation seed production, it is unknown if actors along the value chain would still be willing-to-pay, and if investments can be profitable considering the (highly fluctuating) prices of cassava roots.

The finding that 59% of the farmers acquired NASE14 seed from seed multipliers through cash transactions supports the assumption that there are commercial opportunities for seed of new cassava varieties. Our results indicate that the NASE14-related cash transactions were one-time acquisitions, made during a period of severe disease outbreak; all farmers thereafter multiplied NASE14 for their own use and the majority shared their multiplied seed with fellow farmers. This initial demand for seed of the new variety can give the impression that there is sufficient purchase commitment, but as the variety becomes embedded in the informal seed system, the commercial advantages evaporate (Tripp, 2003). For seed businesses to thrive on new varieties, they would need a steady stream of newly released varieties, which requires linkages breeding and seed programs. In addition, ensuring a constant flow of new varieties could raise other problems: effective campaigns for variety replacement can result in a loss of land races, agrobiodiversity, and in-situ conservation (Pautasso et al., 2013; Thrupp, 2000), and the frequent release of new varieties could complicate choices for farmers (Stone, 2007).

### Farmers access to, and investments in, quality cassava seed

Farmers access to quality cassava seed seemed more variable than their access to new varieties. Many farmers reported a lack of access to quality cassava seed. The definition of ‘quality seed’ was not clarified to them, nor did we ask them for a definition, so the term was open ended and could carry different meanings. Better-off female-headed households (Type 5) were the most likely to perceive themselves as having access to quality seed but used informal sources to acquire it. Most elderly farmers, small-scale farmers, and poor female-headed household perceived themselves to have limited access to quality seed (Type 1,3 and 7). Professional farmers (Type 4) also perceived that they had relatively little access to quality seed although it was this group that made most use of formal sources. This is possibly due to a different or stricter definition of quality seed among professional farmers. Nevertheless, all types of farmers, even professional cassava farmers, most frequently used informal sources to access high quality cassava seed.

Although many farmers reported a lack of access to quality seed, the question remains how this would translate to purchase commitments from seed businesses since its mainly te poorer households who perceive to have limited access. Further research is needed to provide insights in farmers’ willingness-to-pay for quality seed and differences among typologies. Furthermore, for businesses to thrive on the provision of clean seed, seed degeneration patterns need to be understood in order to make predictions on the number of seasons it takes for quality declared and/or certified seed to become advantageous over farm-saved seed. NASE14 seed was generally only acquired once from an off-farm source. Farmers thereafter recycled their material, and the majority reported the quality of their multiplied material had not decreased so far. Seed degeneration rates in farmers’ fields are hard to predict. They depend on many factors such as the environment, management, and variety (Shirima et al., 2019; Thomas-Sharma et al., 2016). Information on seed degeneration should be accompanied by adequate data on yield differences and market prices to show farmers that investments in clean seed are profitable. This information forms the basis of a proper advice on replacement rates, which in turn would be an input in the sales projection for seed busines models: it would define a value proposition and profit formula.

Proper on-farm management practices of vegetative propagated planting material, such as rogueing and positive seed selection, as part of the integrated seed health management approach, are potentially as effective as use of certified seed (Thomas-Sharma et al., 2017). This could allow farmers who, for different reasons, are not able or willing to buy clean seed to produce their own quality seed at lower costs. But, promoting better on-farm management of seed quality would also diminish the demand from seed businesses: fewer farmers would buy clean seed and/or would buy it less frequently. A cassava seed degeneration study in Tanzania showed that varieties have different degeneration patterns: “strong,” “moderate,” “mild,” and “delayed” (Shirima et al., 2019). This insight brings us to another important element of the integrated health management approach (Thomas-Sharma et al., 2016) and a dilemma in the discussion on commercial opportunities for vegetative planting material: market demand for resistant varieties evaporates as they get absorbed in the informal seed system, and demand for clean seed likely decreases due to the milder degeneration patterns of those varieties.

### Tailored business models or an ‘all or none’ approach

Developing farmer typologies and exploring their seed sourcing practices, seed replacement dynamics, and purchasing behavior can assist in designing tailored seed business models. When diversity in seed systems is acknowledged, an integrated seed system development appears to be a suitable approach (Louwaars and De Boef, 2012). In such approach it has been proposed that seed system interventions should not aim to convert all farmers to use commercial seed, but rather to identify those who benefit most from using improved quality seed (Staver et al., 2010). The seed tracing study showed that better-off and more commercial oriented farmers make cash investments in seed, while others rely on seed multiplied by those or otherwise free available seed. This information can support the development of client profiles for commercial seed businesses while simultaneously encouraging informal seed access for farmers who cannot, or do not prefer to, use formal sources.

However, such differentiated approaches also limit the potential clientele of commercial seed businesses. For example, several seed multipliers in our study mentioned they had received their seed from RAB under ‘terms & conditions’, which meant they had to share their multiplied material with fellow farmers, or return a part of their multiplied seed to be distributed. This approach, undoubtedly meant to spur the diffusion of the new varieties, is in contrast with the envisioned business models which would encourage client-farmers to cash purchase from commercial seed businesses. Similarly, integrated seed health approaches suggest reducing farmers’ dependency on external seed sources by, for example, using disease resistant varieties and applying positive selection. These strategies will plausibly affect the commercial demand for clean seed. This does not mean there is no potential for tailored seed business models at all, but rather that they need to be properly coordinated and well-focused. A project estimating the potential for cassava seed businesses in Nigeria, ‘BASICS’, came to similar conclusions and advises an “All or None” approach. This means that all interventions regarding the cassava value chain need to be carried out in alignment in order to avoid one intervention undermining the other (Nitturkar, 2018).

## Conclusions

Commercial seed business models are currently being advocated as a route to developing economically sustainable seed systems providing farmers with materials that overcome a range of production challenges. In this study we developed farmer typologies to inform in the design of seed system interventions, and more specifically to collect data that would assist the development of tailored seed business models. There was a high level adoption of improved varieties among all established farm typologies. Adoption of improved varieties may have happened in response to high disease pressure. Our results indicate that markets for cassava seed emerge to acquire new varieties with desired traits. Cash investments were mainly made by better-off farmers, whereas poorer farmers relied on free access to seed. Many farmers reported a lack of access to quality seed in general, but identified mainly informal sources as potential sources. Since the improved variety NASE14 got introduced in Rwanda early 2015, the majority of farmers used a formal or other off-farm source only once to acquire the variety, and thereafter recycled their material. Farmers generally perceived their recycled material remained of sufficient quality so far.

Based on farmers current seed sourcing strategies, we identified several knowledge gaps that are relevant for the development of viable seed business models. Clarifications and explications on differences between farmers and their willingness-to-pay, the roles of seed degeneration, and cost-benefit analyses seem important requirements for the development of economically sustainable seed business models. To provide cost-benefit analyses that seed businesses could use, it first needs to be defined which parts of the cassava seed value chain remain aid-based or are part of public expenditure, and where commercialization should start. This information can further be used for value propositions and profit formulas of seed business models. In addition, the advocated routes of seed system development have contrasting underlying goals: supporting farmers with free seed or promotion of integrated seed health approaches may affect commercial business models. By acknowledging the differences between farmers, tailored business models might have a high potential impact, but different interventions in the value chain will need to be coordinated to ensure one intervention does not undermine the other.

## Limitations

This study made use of surveys and many variables are self-reported estimations of farmers. Self-reporting is not uncommon despite the biases that may occur. The cassava yields reported by farmers were low (3–4 t/ha). Besides deviations due to self-reported estimations, reported yields are likely lower due to the type of cropping system. It is common in Rwanda to intercrop cassava with other crops such as maize and beans. This would lower the cassava yield/ha but does not directly mean the productivity/plant is low. In a survey from 2007, 78% of the farmers reported they intercropped their cassava (Night et al., 2011), but it cannot be assumed this number was similar in 2020. It is a limitation of this study that the type of cassava cropping system was not recorded, especially as this has influence on disease incidence (Night et al., 2011). Finally, snowball sampling is a useful method for a seed tracing studies, but in practice it turned out to be difficult to follow-up on identified actors. Especially actors far away, or actors with weak social ties between them, are difficult to follow up. This might create a biased image undervaluing the importance of seed exchange among farmers with weak ties or large distances between them.

## Supplemental Material

sj-docx-1-oag-10.1177_00307270211045408 - Supplemental material for Characterizing cassava farmer typologies and their seed sourcing practices to explore opportunities for economically sustainable seed business models in Rwanda Click here for additional data file. Supplemental material, sj-docx-1-oag-10.1177_00307270211045408 for Characterizing cassava farmer typologies and their seed sourcing practices to explore opportunities for economically sustainable seed business models in Rwanda by Fleur Kilwinger, Samuel Mugambi, Rhys Manners, Marc Schut, Silver Tumwegamire, Athanase Nduwumuremyi, Sylvie Bambara and Marthe Paauwe, Conny Almekinders in Outlook on Agriculture

## Notes

1. Cassava is a vegetative propagated crop. Hence cassava planting material is not ‘true seed’ in botanical terms. In Rwanda cassava is mainly multiplied via propagates called ‘stakes’. These are stem cuttings that develop into new plants when planted. Here we use the terms seed, planting material, and stems interchangeably

2. RwF 1,000 equals approximately 1 USD

3. Formal sector standards tend to aim for varietal purity, while, by contrast, farmers may favour a degree of variety heterogeneity, especially when sowing in risk-prone and unpredictable environments. Evidently, assessments of quality vary according to the assessor (Sperling et al., 2008).

4. In the study of Night et al., 2011 large differences among districts were identified (15% vs. 100% of farmers growing only local varieties in Bugesera and Ruhango respectively), which were not identified in this study (data not presented).

## Annex 1: Additional variety information

**Table A1.** Varieties identified as grown by farmers, their year of official release in Rwanda and the percentage of farmers growing them (n = 390).

	Variety name(s)	Type and official name	Official year of release (approximate)	Grown by % of farmers
1	NASE14, Tubura, Umweru, Imyeru, Bizigira, RAB	Improved (NASE 14)	2018	51%
2	Macadamia	Improved (MM96/8299)	2021	44%
3	RAB	Improved varieties	2018	3%
4	NAROCAS1	Improved (TZ130)	2018	1%
5	Kizere	Improved (I92/0057)	2006	8%
6	Nyiragatare, Mbakungahaze	Improved (95/NA/00063)	2006	4%
7	Mavoka, Mavuta Umuhondo	Improved (MM96/0287)	2009	4%
8	Mbagarumbise	Improved (MH95/0414)	2006	1%
9	Serura, Seruruseke	Improved (MM96/5280)	2009	1%
10	Ndamirabana	Improved (TME 14)	2006	<1%
11	Eala 07, Gapfunsi, Gitaminsi	Improved Eala 07	1975	15%
12	Creolinha, Gafuni, Rushyirwinkuba, Rutanihisha, Zanagafuni	Improved Creolinha	1985	8%
13	Amuri, Amurine, Mure	Improved MM96/9688	From trial	6%
14	Maguruyinkware, Buguru, Bwinkware	Improved Maguruyinkware	1985	3%
15	Kibombwe Buryohe, maryohe, Iminayelo, Kibomwe, Iminayiro	Improved Kibombwe	1985	2%
16	Bukarasa	Improved Bukarasa	1985	1%
17	Imizungu	Improved	Not known	1%
18	Imitanzaniya	Improved	Not known	<1%
19	Nyirakarasi	Landrace	-	8%
20	Gahene	Landrace	-	6%
21	Nyabushabure	Landrace	-	3%
22	Kigoma	Landrace	-	2%
23	Gacyacyali	Landrace	-	2%
24	Imiribwa	Landrace	-	2%
25	Rwakarori	Landrace	-	2%
26	Nyiramasibo	Landrace	-	2%
27	Imicyari	Landrace	-	1%
28	Itukura	Landrace	-	1%
29	Kavumu	Landrace	-	1%
30	Kicaro	Landrace	-	1%
31	Amaso manini	Landrace	-	<1%
32	Charlotti	Landrace	-	<1%
33	Cyiso	Landrace	-	<1%
34	Iminyarwanda	Landrace	-	<1%
35	Makesa	Landrace	-	<1%
36	Manoyinanga	Landrace	-	<1%
37	Mbundanyi	Landrace	-	<1%
38	Mushedire	Landrace	-	<1%
39	Nyiramabuye	Landrace	-	<1%
40	Sinihaniza	Landrace	-	<1%
41	Umugande	Landrace	-	<1%
42	Umunanira	Landrace	-	<1%
43	Butukura	Landrace	-	<1%
44	Umubombwe	Landrace	-	<1%
45	Pakiya	Landrace	-	<1%
46	Rutare	Landrace	-	<1%

## References

[bibr1-00307270211045408] Alicai T Omongo CA Maruthi MN , et al. (2007) Re-emergence of cassava brown streak disease in Uganda. Plant Disease 91: 24–29. 30781061 10.1094/PD-91-0024

[bibr2-00307270211045408] Almekinders CJM Ronner E van Heerwaarden J (2020) Tracing legume seed diffusion beyond demonstration trials: An exploration of sharing mechanisms. Outlook on Agriculture 49(1): 29–38. 32641869 10.1177/0030727020907646 PMC7307448

[bibr3-00307270211045408] Almekinders CJM Walsh S Jacobsen KS , et al. (2019) Why interventions in the seed systems of roots, tubers and bananas crops do not reach their full potential. Food Security 11(1): 23–42.

[bibr4-00307270211045408] Alvarez S Timler CJ Michalscheck M , et al. (2018) Capturing farm diversity with hypothesis-based typologies: An innovative methodological framework for farming system typology development. PLoS One 13(5): e0194757. 29763422 10.1371/journal.pone.0194757 PMC5953459

[bibr5-00307270211045408] Barker I Jones R Klauser D (2021) Smallholder seed systems for sustainability. In: Klauser D Robinson M (eds) The Sustainable Intensification of Smallholder Farming Systems. Burleigh Dodds Science Publishing: Cambridge, 1–18.

[bibr6-00307270211045408] Bartle B Maredia MK (2019) The Effect of Quality Signaling and Trust on Willingness to Pay for Potato Planting Material: A Research Study in Kenya. Selected Paper prepared for presentation at the *Agricultural & Applied Economics Association Annual Meeting*, Atlanta, GA, 21–23 July 2019.

[bibr7-00307270211045408] Bentley JW Andrade-Piedra JL Demo P , et al. (2018) Understanding root, tuber, and banana seed systems and coordination breakdown: A multi-stakeholder framework. Journal of Crop Improvement 32(5): 599–621.

[bibr8-00307270211045408] Bentley JW Nitturkar H Obisesan D , et al. (2020) Is there a space for medium-sized cassava seed growers in Nigeria? Journal of Crop Improvement 34(1): 16.

[bibr9-00307270211045408] Boadu P Aidoo R Ohene-Yankyera K , et al. (2019) A latent class modelling approach to evaluating farmers’ preferences for pona seed yam certification systems and their willingness to pay in Ghana. International Journal of Agricultural Extension and Rural Development Studies 6(1): 1–25.

[bibr10-00307270211045408] Campos H (2021) ‘The quest for innovation: Addressing user needs and value creation’. In: Campos H (eds) The Innovation Revolution in Agriculture: A Roadmap to Value Creation. Cham: Springer, 1–29.

[bibr11-00307270211045408] Coomes OT McGuire SJ Garine E , et al. (2015) Farmer seed networks make a limited contribution to agriculture? Four common misconceptions. Food Policy 56: 41–50.

[bibr12-00307270211045408] CtEH (2021) *Accelerating the delivery of quality seed from breeding investments made by the Crops to End Hunger (CtEH) initiative through economically sustainable seed systems.* White Paper commissioned by Crops to End Hunger, February 2021. Available at: https://www.syngentafoundation.org/sites/g/files/zhg576/f/2021/03/23/white_paper2021final.pdf (accessed 18 August 2021).

[bibr13-00307270211045408] Delêtre M Lett JM Sulpice R , et al. (2021) Kinship networks of seed exchange shape spatial patterns of plant virus diversity. Nature Communications 12(1): 1–10. 10.1038/s41467-021-24720-6 PMC8302746 34301941

[bibr14-00307270211045408] Donovan J Rutsaert P Mausch K , et al. (2021) ‘Strengthening seed value chains: Persistent challenges and ways forward’. Slide deck prepared as input for the *One-CGIAR Strategy on Seed Systems Development*. Available at: https://pimcgiarorg/cgiar-coe-seed-systems-development/references-and-outputs/ (accessed 4 June 2021).

[bibr15-00307270211045408] Dray S Dufour AB (2007) The ade4 package: Implementing the duality diagram for ecologists. Journal of Statistical Software 22(4): 1–20.

[bibr16-00307270211045408] Garrett KA (2021) Impact network analysis and the INA R package: Decision support for regional management interventions. Methods in Ecology and Evolution 00: 1–14.

[bibr17-00307270211045408] Gibson RW Mwanga ROM Namanda S , et al. (2009) Review of Sweetpotato Seed Systems in East and Southern Africa. International Potato Center (CIP). Lima: Integrated crop management working paper. pp. 48.

[bibr18-00307270211045408] Goodman LA (1961) Snowball sampling. The annals of mathematical statistics: 148–170.

[bibr19-00307270211045408] Gower JC (1971) A general coefficient of similarity and some of its properties. Biometrics 27: 857–874.

[bibr20-00307270211045408] Hammond J Fraval S Van Etten J , et al. (2017) ‘The rural household multi-indicator survey (RHoMIS) for rapid characterisation of households to inform climate smart agriculture interventions: Description and applications in east Africa and Central America. Agricultural Systems 151: 225–233.

[bibr21-00307270211045408] Hammond J Rosenblum N Breseman D , et al. (2020) Towards actionable farm typologies: Scaling adoption of agricultural inputs in Rwanda. Agricultural Systems 183: 102857.

[bibr22-00307270211045408] IITA (2015) *Partners discuss management strategies for cassava diseases in Rwanda.* IITA Bulletin, no. 2293, 15-18 September 2015. Available at: http://oar.icrisat.org/9146/1/IITA%20Bulletin_2293LOWRES.pdf (accessed 18 August 2021).

[bibr23-00307270211045408] Jolliffe IT (2002) Principal components in regression analysis. In: Principal Component Analysis. Springer Series in Statistics. New York: Springer. pp 167–198.

[bibr24-00307270211045408] Kawuki SR Kaweesi T Esuma W , et al. (2016) Eleven years of breeding efforts to combat cassava brown streak disease. Breeding Science 66(4): 560–571. 27795681 10.1270/jsbbs.16005 PMC5010303

[bibr25-00307270211045408] Kilwinger FBM Buddenhagen CE (2021) ‘User guide to seed tracing’. Lima (Peru) *CGIAR Research Program on Roots, Tubers and Bananas (RTB). RTB User Guide No 2021-1* Available at: www.rtb.cgiar.org (accessed 4 June 2021).

[bibr26-00307270211045408] Kilwinger FBM Marimo P Rietveld AM , et al. (2020) ‘Not only the seed matters: Farmers’ perceptions of sources for banana planting materials in Uganda’. Outlook on Agriculture 49(2): 119–132. 33281230 10.1177/0030727020930731 PMC7684323

[bibr27-00307270211045408] Kuivanen KS Alvarez S Michalscheck M , et al. (2016) Characterising the diversity of smallholder farming systems and their constraints and opportunities for innovation: A case study from the northern region, Ghana. NJAS-Wageningen Journal of Life Sciences 78: 153–166.

[bibr28-00307270211045408] Legg JP Jeremiah SC Obiero HM , et al. (2011) Comparing the regional epidemiology of the cassava mosaic and cassava brown streak virus pandemics in Africa. Virus Research 159(2): 161–170. 21549776 10.1016/j.virusres.2011.04.018

[bibr29-00307270211045408] Legg JP Okao-Okuja G Mayala R , et al. (2001) Spread into Rwanda of the severe cassava mosaic virus disease pandemic and the associated Uganda variant of East African cassava mosaic virus (EACMV-Ug). Plant Pathology 50(6): 796.

[bibr30-00307270211045408] Lopez-Ridaura S Frelat R van Wijk MT , et al. (2018) Climate smart agriculture, farm household typologies and food security: An ex-ante assessment from eastern India. Agricultural Systems 159: 57–68. 29302131 10.1016/j.agsy.2017.09.007 PMC5738964

[bibr31-00307270211045408] Louwaars NP De Boef WS (2012) Integrated seed sector development in Africa: A conceptual framework for creating coherence between practices, programs, and policies. Journal of Crop Improvement 26(1): 39–59.

[bibr32-00307270211045408] Maggidi IM , 2019. Cassava Value Chain: Willingness To Pay for Improved Cassava Planting Material in Coastal and Lake Victoria areas of Tanzania . MSc Thesis. Sokoine University of Agriculture, Tanzania.

[bibr33-00307270211045408] Manyong VM Dixon AGO Makinde KO , et al. (2000) ‘The contribution of IITA-improved cassava to food security in sub-Saharan Africa: An impact study’. Available at: http://www fao org/docs/eims/upload/166448/ (accessed 4 June 2021).

[bibr34-00307270211045408] Maredia MK Shupp R Opoku E , et al. (2019) Farmer perception and valuation of seed quality: Evidence from bean and cowpea seed auctions in Tanzania and Ghana. Agricultural Economics 50(4): 495–507.

[bibr35-00307270211045408] McGuire SJ (2008) Securing access to seed: Social relations and sorghum seed exchange in eastern Ethiopia. Human Ecology 36(2): 217–229.

[bibr36-00307270211045408] McGuire SJ Sperling L (2016) Seed systems smallholder farmers use. Food Security 8(1): 179–195.

[bibr37-00307270211045408] Namanda S Gibson RW Sindi K (2011) Sweetpotato seed systems in Uganda, Tanzania, and Rwanda. Journal of Sustainable Agriculture 35: 870–884.

[bibr38-00307270211045408] Night G Asiimwe P Gashaka G , et al. (2011) Occurrence and distribution of cassava pests and diseases in Rwanda. Agriculture, Ecosystems & Environment 140(3–4): 492–497.

[bibr39-00307270211045408] Nitturkar H (2018) “All or None” Approach to Developing a Cassava Seed System in Nigeria. Available at: https://www.marketlinks.org/blogs/all-or-none-approach-developing-cassava-seed-system-nigeria (accessed 4 June 2021).

[bibr40-00307270211045408] Okonya SJ Ocimati W Nduwayezu A , et al. (2019) Farmer reported pest and disease impacts on root, tuber, and banana crops and livelihoods in Rwanda and Burundi. Sustainability 11: 1592.

[bibr41-00307270211045408] Patil LB Legg PJ Kanju E , et al. (2015) Cassava brown streak disease: A threat to food security. Africa Journal of General Virology 96: 956–968. 10.1099/vir.0.000014 26015320

[bibr42-00307270211045408] Pautasso M Aistara G Barnaud A , et al. (2013) Seed exchange networks for agrobiodiversity conservation. A review. Agronomy for sustainable development 33(1): 151–175.

[bibr43-00307270211045408] Rachkara P Phillips DP Kalule SW , et al. (2017) Innovative and beneficial informal sweetpotato seed private enterprise in northern Uganda. Food Security 9(3): 595–610. 32968464 10.1007/s12571-017-0680-4 PMC7473088

[bibr44-00307270211045408] R Core Team (2019) *R: A language and environment for statistical computing*. R Foundation for Statistical Computing. Vienna, Austria. Available at: https://www.R-project.org (accessed 4 June 2021).

[bibr45-00307270211045408] Reynolds A Richards G De la Iglesia B , et al. (1992) Clustering rules: A comparison of partitioning and hierarchical clustering algorithms. Journal of Mathematical Modelling and Algorithms 5: 475–504.

[bibr46-00307270211045408] Rohrbach DD Mashingaidze AB Mudhara M (2005) Distribution of Relief Seed and Fertilizer in Zimbabwe: Lessons From the 2003/04 Season. PO Box 776, Bulawayo, Zimbabwe: ICRISAT. and Rome, Italy: FAO. 36 pp.

[bibr47-00307270211045408] Shirima RR Maeda DG Kanju EE , et al. (2019) Assessing the degeneration of cassava under high-virus inoculum conditions in coastal Tanzania. Plant Disease 103(10): 2652–2664. 31322490 10.1094/PDIS-05-18-0750-RE PMC7779971

[bibr48-00307270211045408] Sperling L Boettiger S Barker I (2013) *Integrating seed systems.* Planning for scale brief no. 3. AgPartnerXChange 2013. Available at: https://seedsystem.org/wp-content/uploads/2014/03/Integrating-Seed-Systems-.pdf (accessed 17 August 2021).

[bibr49-00307270211045408] Sperling L Cooper HD Remington T (2008) Moving towards more effective seed aid. The Journal of Development Studies 44(4): 586–612.

[bibr50-00307270211045408] Staver C Van den Bergh I Karamura E , et al. (2010) Targeting actions to improve the quality of farmer planting material in bananas and plantains - building a national priority-setting framework. Tree and Forestry Science and Biotechnology 4(1): 1–10.

[bibr51-00307270211045408] Stone GD (2007) Agricultural deskilling and the spread of genetically modified cotton in Warangal. Current Anthropology 48(1): 67–103.

[bibr52-00307270211045408] Tadesse Y Almekinders CJM Schulte RP , et al. (2017) ‘Tracing the seed: Seed diffusion of improved potato varieties through farmers’ networks in Chencha, Ethiopia’. Experimental Agriculture 53(4): 481.

[bibr53-00307270211045408] Thiele G (1999) Informal potato seed systems in the Andes: Why are they important and what should we do with them? World Development 27(1): 83–99.

[bibr54-00307270211045408] Thomas-Sharma S Abdurahman A Ali S , et al. (2016) Seed degeneration in potato: The need for an integrated seed health strategy to mitigate the problem in developing countries. Plant Pathology 65(1): 3–16.

[bibr55-00307270211045408] Thomas-Sharma S Andrade-Piedra J Carvajal Yepes M , et al. (2017) A risk assessment framework for seed degeneration: Informing an integrated seed health strategy for vegetatively propagated crops. Phytopathology 107(10): 1123–1135. 28545348 10.1094/PHYTO-09-16-0340-R

[bibr56-00307270211045408] Thrupp LA (2000) Linking agricultural biodiversity and food security: The valuable role of agrobiodiversity for sustainable agriculture. International Affairs 76(2): 265–281. 18383639 10.1111/1468-2346.00133

[bibr57-00307270211045408] Tripp R (2003) ‘How to cultivate a commercial seed sector’. In: symposium on *Sustainable Agriculture in the Sahel*, Bamako, Mali, 1-5 December 2003.

[bibr58-00307270211045408] Tripp R Rohrbach D (2001) Policies for African seed enterprise development. Food Policy 26(2): 147–161.

[bibr59-00307270211045408] Tumwegamire S Kanju E Legg J , et al. (2018) Exchanging and managing in-vitro elite germplasm to combat cassava brown streak disease (CBSD) and cassava mosaic disease (CMD) in Eastern and Southern Africa. Food Security 10(2): 351–368. 33365104 10.1007/s12571-018-0779-2 PMC7705177

