# Supplementary material for: Characterizing cassava farmer typologies and their seed sourcing practices to explore opportunities for economically sustainable seed business models in Rwanda
Source: Outlook Agric. 2021 Nov 30;50(4):441–54. doi: 10.1177/00307270211045408 (PMC8637389; doi:10.1177/00307270211045408)
Supplement: sj-docx-1-oag-10.1177_00307270211045408 - Supplemental material for Characterizing cassava farmer typologies and their seed sourcing practices to explore opportunities for economically sustainable seed business models in Rwanda [file sj-docx-1-oag-10.1177_00307270211045408.docx]

# Annex 1: Additional variety information

| Table 1. Varieties identified as grown by farmers, their year of official release in Rwanda and the percentage of farmers growing them (n=390). | | | | |
| --- | --- | --- | --- | --- |
|  | Variety name(s) | Type and official name | Official year of release (approximate) | Grown by % of farmers |
| 1 | NASE14, Tubura, Umweru, Imyeru, Bizigira, RAB | Improved (NASE 14) | 2018 | 51% |
| 2 | Macadamia | Improved (MM96/8299) | 2021 | 44% |
| 3 | RAB | Improved varieties | 2018 | 3% |
| 4 | NAROCAS1 | Improved (TZ130) | 2018 | 1% |
| 5 | Kizere | Improved (I92/0057) | 2006 | 8% |
| 6 | Nyiragatare, Mbakungahaze | Improved (95/NA/00063) | 2006 | 4% |
| 7 | Mavoka, Mavuta Umuhondo | Improved (MM96/0287) | 2009 | 4% |
| 8 | Mbagarumbise | Improved (MH95/0414) | 2006 | 1% |
| 9 | Serura, Seruruseke | Improved (MM96/5280) | 2009 | 1% |
| 10 | Ndamirabana | Improved (TME 14) | 2006 | <1% |
| 11 | Eala 07, Gapfunsi, Gitaminsi | Improved Eala 07 | 1975 | 15% |
| 12 | Creolinha, Gafuni, Rushyirwinkuba, Rutanihisha, Zanagafuni | Improved Creolinha | 1985 | 8% |
| 13 | Amuri, Amurine, Mure | Improved MM96/9688 | From trial | 6% |
| 14 | Maguruyinkware, Buguru, Bwinkware | Improved Maguruyinkware | 1985 | 3% |
| 15 | Kibombwe Buryohe, maryohe, Iminayelo, Kibomwe, Iminayiro | Improved Kibombwe | 1985 | 2% |
| 16 | Bukarasa | Improved Bukarasa | 1985 | 1% |
| 17 | Imizungu | Improved | Not known | 1% |
| 18 | Imitanzaniya | Improved | Not known | <1% |
| 19 | Nyirakarasi | Landrace | - | 8% |
| 20 | Gahene | Landrace | - | 6% |
| 21 | Nyabushabure | Landrace | - | 3% |
| 22 | Kigoma | Landrace | - | 2% |
| 23 | Gacyacyali | Landrace | - | 2% |
| 24 | Imiribwa | Landrace | - | 2% |
| 25 | Rwakarori | Landrace | - | 2% |
| 26 | Nyiramasibo | Landrace | - | 2% |
| 27 | Imicyari | Landrace | - | 1% |
| 28 | Itukura | Landrace | - | 1% |
| 29 | Kavumu | Landrace | - | 1% |
| 30 | Kicaro | Landrace | - | 1% |
| 31 | Amaso manini | Landrace | - | <1% |
| 32 | Charlotti | Landrace | - | <1% |
| 33 | Cyiso | Landrace | - | <1% |
| 34 | Iminyarwanda | Landrace | - | <1% |
| 35 | Makesa | Landrace | - | <1% |
| 36 | Manoyinanga | Landrace | - | <1% |
| 37 | Mbundanyi | Landrace | - | <1% |
| 38 | Mushedire | Landrace | - | <1% |
| 39 | Nyiramabuye | Landrace | - | <1% |
| 40 | Sinihaniza | Landrace | - | <1% |
| 41 | Umugande | Landrace | - | <1% |
| 42 | Umunanira | Landrace | - | <1% |
| 43 | Butukura | Landrace | - | <1% |
| 44 | Umubombwe | Landrace | - | <1% |
| 45 | Pakiya | Landrace | - | <1% |
| 46 | Rutare | Landrace | - | <1% |
